# Validation of a musculoskeletal model for simulating muscle mechanics and energetics during diverse human hopping tasks

**DOI:** 10.1098/rsos.230393

**Published:** 2023-10-25

**Authors:** Luke N. Jessup, Luke A. Kelly, Andrew G. Cresswell, Glen A. Lichtwark

**Affiliations:** School of Human Movement and Nutrition Sciences, Centre for Sensorimotor Performance, The University of Queensland, Brisbane, Queensland, Australia

**Keywords:** biomechanics, computation, locomotion, ultrasound, electromyography, dataset

## Abstract

Computational musculoskeletal modelling has emerged as an alternative, less-constrained technique to indirect calorimetry for estimating energy expenditure. However, predictions from modelling tools depend on many assumptions around muscle architecture and function and motor control. Therefore, these tools need to continue to be validated if we are to eventually develop subject-specific simulations that can accurately and reliably model rates of energy consumption for any given task. In this study, we used OpenSim software and experimental motion capture data to simulate muscle activations, muscle fascicle dynamics and whole-body metabolic power across mechanically and energetically disparate hopping tasks, and then evaluated these outputs at a group- and individual-level against experimental electromyography, ultrasound and indirect calorimetry data. Comparing simulated and experimental outcomes, we found weak to strong correlations for peak muscle activations, moderate to strong correlations for absolute fascicle shortening and mean shortening velocity, and strong correlations for gross metabolic power. These correlations tended to be stronger on a group-level rather than individual-level. We encourage the community to use our publicly available dataset from SimTK.org to experiment with different musculoskeletal models, muscle models, metabolic cost models, optimal control policies, modelling tools and algorithms, data filtering etc. with subject-specific simulations being a focal goal.

## 1. Introduction

Determining the metabolic energy demands of specific movements is important for exercise prescription, for the design of assistive devices, and for understanding why we choose to move the way that we do. While it is possible to determine energy expenditure through direct or indirect calorimetry, these techniques require repetitive motion, typically at steady-state, over prolonged periods of time [1], and the set-ups are high-priced and usually lack portability. Alternatively, we can predict energy expenditure from mechanical function, which is popularly done via computational musculoskeletal modelling tools and is often more accessible in practice [2–5]. However, these tools depend on many assumptions around muscle architecture and function and motor control, and thus, need to be validated as this area continues to grow.

Musculoskeletal models typically estimate rates of energy consumption based on the states (e.g. activation, length, velocity) of Hill-type muscle models required to produce a specific movement pattern [6–10]. While such models are validated against direct measures of muscle energy consumption from isolated muscle preparations, the prediction of energy consumption for whole-body tasks requires accurate estimates of the mechanical properties of each muscle (e.g. fibre and tendon length, tendon stiffness) and the subsequent prediction of the muscle states to generate appropriate forces. Typically, the muscle redundancy problem (i.e. having more muscles than degrees of freedom of joints) requires that an optimization algorithm and some objective criteria (e.g. minimize muscle activation or stress) is used to solve the muscle states of each actuator. A general lack of certainty in both individual muscle properties and the optimization means that there is currently no ‘standardized' model of musculoskeletal structure and function that has been shown to be generalizable across subjects or movement tasks.

Given these uncertainties, it is unsurprising that varying levels of correlation are reported between experimental and simulated muscle activations and fascicle dynamics, even for simple walking tasks. For instance, Delabastita *et al*. [11] performed a study that aimed to individualize muscle parameters in models using ultrasound measurements to improve the fit between experimental and simulated muscle fascicle lengths and muscle activations across walking speeds. Between the groups that were analysed, they reported a range of moderate to strong correlations between experimental and simulated medial gastrocnemius (GM) fascicle length time-series data, and a range of weak to moderate correlations for lateral gastrocnemius (GL), soleus (SOL) and tibialis anterior (TA) muscle activation time-series data. Importantly, they noted that these correlations were even more variable on an individual level, which, not for the first time, brings into question current modelling approaches' sensitivity to inter-individual differences [12–15].

Despite variance between modelled and measured muscle states, moderate to strong correlations are reported between modelled and measured energy expenditure for both group and individual comparisons across tasks like walking. For instance, Koelewijn *et al*. [16] performed a study that aimed to compare the metabolic cost simulated by different cost models to that measured experimentally across different walking speeds and slopes. Across all trials and all models tested, they reported strong correlations between experimental and modelled metabolic power. However, they and Miller [17] note that the absolute performance of these cost models is variable, resulting from the equations used and/or inaccuracies in the model inputs (e.g. muscle mechanics).

Most model development and validation studies have been reserved to select gait conditions (e.g. walking or running across limited speeds; [11,16–19]). Moreover, to our knowledge, no study has tested movements that might have drastically different force requirements. This could be problematic because mechanics and energetics can vary markedly with subtle and broad changes to locomotor conditions. Therefore, we aimed to simulate muscle activations, muscle fascicle dynamics and whole-body metabolic power using popular modelling software [20] across mechanically and energetically disparate hopping tasks, and then to evaluate the simulated data at a group- and individual-level against data obtained experimentally. We examined a hopping movement, rather than gait, because we could more easily manipulate the task in ways that more acutely changed the energetic demands. Thus, we could assess the ability to model energy consumption under a wide range of muscle function conditions that are probably more generalizable to everyday tasks, including but not exclusive to gait.

## 2. Material and methods

### 2.1. Data collation

The experimental data used to generate and validate simulations of hopping is from an earlier study we conducted that is described in detail elsewhere [21]. To achieve the aims of the current study, we conducted analyses of eight participants (five male, three female), age 26 ± 2 (mean ± s.d.) years, height 174 ± 12 cm, mass 72 ± 18 kg. The remaining four participants from the earlier study [21] were removed from this study because they had erroneous motion capture data for one or more of the seven testing conditions mentioned below.

#### 2.1.1. Experimental data

Briefly, we selected 7 of the 19 different hopping conditions that participants performed. This subset of data was chosen because it spanned across the variety of hop heights and frequencies of those 19 conditions, thereby encompassing the whole range of mechanics and metabolic rates. This was also done to reduce the significant computational time that it would have taken to simulate those 19 conditions across our eight participants. Specifically, we selected three height-constrained conditions: low height (LH) (0.06 m), medium height (MH) (0.11 m) and high height (HH) (0.16 m), with hop frequency unconstrained; and four frequency-constrained conditions: low frequency (LF) (1.8 Hz), low-medium frequency (LMF) (2.2 Hz), medium-high frequency (MHF) (2.6 Hz) and high frequency (HF) (3.0 Hz), with hop height unconstrained.

Ground reaction force (GRF) data were collected at 1000 Hz from two force plates (OR6–7, AMTI, MA, USA), one positioned under each foot. An 11 camera motion analysis system (Oqus, Qualisys, AB, Sweden) was used to capture the position of 43 reflective markers at 200 Hz and was synchronized with the GRF data. Markers were placed on anatomical landmarks on both feet, shanks, and thighs, and on the pelvis and torso. B-mode ultrasound (LZ 7.5/60/96Z, Telemed, Vilnius, Lithuania) was used to image GL, SOL and vastus lateralis (VL) muscle fascicles at 160 Hz, and was synchronized to the GRF and motion capture data using an external trigger. Surface electromyography (EMG) (MA422, Motion Lab Systems, CA, USA) was collected for GL, GM, SOL, TA, VL, rectus femoris (RF) and biceps femoris (BF) in Qualisys Track Manager at 2000 Hz. Oxygen consumption and carbon dioxide elimination were measured with a portable spirometry system (Metamax 3B; Cortex, Leipzig, Germany).

Automated tracking software was used to determine fascicle lengths from the ultrasound data [22,23]. Fascicle length changes during each hop cycle were cropped from ground contact through to the end of the subsequent flight phase, and time-series averaged across five hops. In order to compare across participants, fascicle lengths were normalized to the respective mean length across all the trials that each participant performed, termed mean fascicle length (*L*_M_). Fascicle shortening was calculated as the difference between the maximum fascicle length during ground contact and the minimum fascicle length immediately following ground contact. Fascicle shortening velocity (*L*_M_._S_^−1^) was calculated over the same period as the derivative of the normalized length signal.

EMG data were pre-amplified with 1000-times gain, and hardware filtered with a bandwidth of 10–2000 Hz. Subsequently, DC offsets were removed from the data, and the signal was high-pass filtered at 25 Hz, then rectified, and then finally filtered with zero-lag by applying a second order Butterworth filter with a 10 Hz cut-off frequency forwards and backwards in time, producing an envelope [24]. For each participant, EMG signals were cropped from ground contact through to the end of the subsequent flight phase, and then time-series averaged across five hops. Each signal was then normalized to the mean EMG across the hop cycle of all trials being analysed from the same respective session, making the data suitable for deciphering relative changes in peak EMG between sessions.

Oxygen consumption data from the final 2 min period of each trial was averaged and then converted to gross metabolic power using standard equations [25].

#### 2.1.2. Simulated data

GRF and motion capture data were digitally exported via Qualisys software via the C3D file format for use in OpenSim (v4.2.0). We selected the open-source model published by Lai *et al*. [26] as the base model for our analysis. This model, which is comprised 80 Hill-type muscle-tendon units, is a refined version of the model by Rajagopal *et al*. [18], where modifications were made to reduce excessive passive forces when simulating movements that involve substantial hip and knee flexion. Because hopping meets this criteria, and that we found high passive forces to be a problem when piloting other popular models on our data [18,19], we decided that the Lai *et al*. [26] model was most suitable. We scaled this model using OpenSim's scale tool, which calculates scaling factors by dividing participant-specific marker distances by the corresponding distances on the generic model. Body kinematics during hopping were then determined using OpenSim's inverse kinematics tool, which used a weighted least-squares fit of the generic markers rigidly attached to the scaled model to the experimental markers. Body kinematics and GRF data were then provided to OpenSim's inverse dynamics tool where they were filtered at 15 Hz and used to determine body kinetics. Note, OpenSim's filter did not filter the time vector, nor did it affect the phase of the signals. Ankle, knee and hip kinematics and kinetics were visually inspected against hopping data reported in the literature, and residual forces were checked to be less than 5% of the maximum GRF magnitude, as per recommendations [27].

The muscle properties of our scaled models were then adjusted for use in OpenSim Moco (v0.4.0) [28]. Moco uses direct collocation to solve optimal control problems, in our case, taking the body kinematics and kinetics that we prescribed and using Moco's default cost function of minimizing the sum of square controls to solve for muscle excitations and fascicle length changes. Moreover, Moco uses the DeGrooteFregly2016Muscle [29], therefore all muscles were converted to this muscle model type. Nineteen degrees of freedom (six at the pelvis, five in each leg and three in the upper body) were considered in the model, discounting the metatarsophalangeal and subtalar joints which were locked to prevent instability that otherwise caused the muscles that spanned those joints to be overactive in simulation. We then went through a systematic process of manually adjusting DeGrooteFregly2016Muscle properties within the Lai *et al.* [26] model (i.e. optimal fascicle lengths, tendon slack lengths, tendon stiffnesses, maximum isometric forces) to see whether we could improve the agreement (i.e. both visually and using the statistical analyses described below) between our experimental data and the resultant muscle excitations and activations and fascicle length changes from Moco. We did this for three of the eight subjects that ranged in mass (65–87 kg). We also tried using an automated approach to adjusting muscle properties [14]. With adjustments to the muscle properties, we found no clear improvements in correlation between simulated and experimental activation and fascicle dynamics. Between participants, we decided to adjust the models' GL, SOL and VL optimal fascicle lengths to the respective mean fascicle lengths to which we normalized our experimental data and adjust the tendon slack lengths to account for these changes (thereby maintaining a similar joint angle at which muscle fascicle length first starts to increase when the joint is passively rotated), and to scale all of the models' muscle maximum isometric forces based on the mass of the participant relative to the mass of the generic Lai *et al*. [26] model.

Per trial, the particular hop cycle that we chose to simulate in Moco was based visually on (i) the symmetry (i.e. in magnitude and shape) between left and right ankle, knee and hip moments, and (ii) the magnitude and shape of these moments compared to the average of all hop cycles collected for that trial. For each simulated hop cycle (i.e. from ground contact through to the end of the subsequent flight phase), fascicle lengths and muscle activations were retrieved for the same muscles measured experimentally.

Simulated fascicle lengths were normalized to the same values as the experimental data (*L*_M_), and fascicle shortening and shortening velocity was calculated the same way mentioned previously. We took specific interest in the magnitudes of fascicle shortening and fascicle shortening velocity because these are what predominantly drive increases in muscle metabolic cost [30–32]. As per the process of normalizing the experimental EMG, simulated muscle activations were normalized to the mean simulated muscle activation within their respective session—this meant that our analysis could be performed independent of scaling factors (i.e. maximum voluntary contraction in EMG or maximum isometric force in model). We were able to compare peak muscle activations since the hopping conditions that we tested drastically differed in their force requirements. Analysing these scalar metrics ensured that the results were not affected by time-shifts that we observed between experimental and simulated data.

To compare muscle activations, we assume that the filtered excitation signal measured directly from the muscle (i.e. enveloped EMG) is analogous to the calculated activation within the muscle model. While we could instead compare the excitation signal from the muscle model, the excitation-activation transformation effectively acts as a low-pass filter, and thus, the comparison that we make is reasonable. We discuss limitations of this approach in the discussion.

To estimate metabolic energy expenditure from the simulations, we used a cost model developed by Umberger *et al*. [10,33], with some modifications [5]. To employ the cost model, the results from Moco were used as inputs to OpenSim's Umberger2010MuscleMetabolicsProbe through the OpenSim probe reporter tool. To compute gross whole-body metabolic power from the cost model, we summed the outputted rate of energy consumption across all muscles, plus the outputted whole-body mass-specific basal rate, and then integrated the resulting whole-body rate over the hop cycle and divided by the duration of the hop cycle.

### 2.2. Statistics

#### 2.2.1. Group analysis

Experimental and simulated peak muscle activations, absolute fascicle shortening, mean fascicle shortening velocities and gross metabolic power were grouped according to the experimental condition that they belonged to: LH (2.62 ± 0.25 Hz, 0.09 ± 0.01 m); MH (2.16 ± 0.19 Hz, 0.14 ± 0.01 m); HH (1.85 ± 0.13 Hz, 0.19 ± 0.01 m); LF (1.89 ± 0.13 Hz, 0.11 ± 0.02 m); LMF (2.24 ± 0.04 Hz, 0.10 ± 0.02 m); MHF (2.62 ± 0.05 Hz, 0.09 ± 0.02 m); or HF (2.98 ± 0.07 Hz, 0.08 ± 0.01 m). For each metric, the experimental and simulated data were averaged across all of the values for each hopping condition. The coefficient of determination (*R*^2^) was then computed between the mean experimental values and mean simulated values to determine how well the experimental data and simulated data correlated on a group-level (G). We considered *R^2^* values of ≤0.35 as weak correlations, 0.36–0.69 as moderate correlations, 0.70–0.89 as strong correlations, and ≥0.90 as very strong correlations [34].

#### 2.2.2. Individual analysis

For each metric, all experimental and corresponding simulated data was provided to a linear mixed-effects model. In these models, experimental data were used as the response variable, simulated data were used as the fixed effect, and participant was used as a random effect. This, combined with an interaction term, allowed us to account for random intercepts and random slopes in each participant's data. The formula can be written in Wilkinson notation as:

experimental=simulated+(simulated|participant).

The *R*^2^ was then calculated between the fitted (modelled) data and response (original) data to determine how well the experimental and simulated data correlated on an individual-level (I).

## 3. Results

### 3.1. Muscle activations

**Figure 1.**
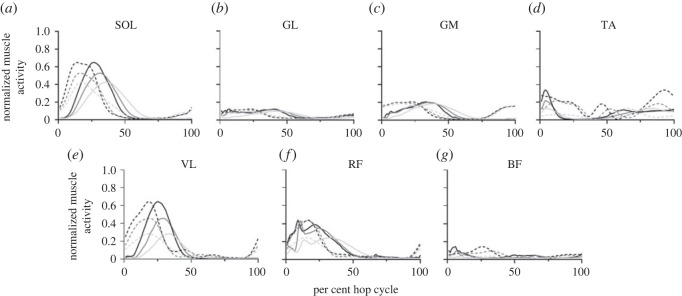
Time-series plots of the normalized experimental (dotted) and simulated (solid) muscle activity of three conditions (LH, MH, HH—from lightest to darkest) for SOL (*a*), GL (*b*), GM (*c*), TA (*d*), VL (*e*), RF (*f*), and BF (*g*). The data have been averaged across all participants for each condition. Experimental muscle activity is range normalized to the minimum and maximum value of the respective simulated muscle activity.

The simulated muscle activations generally appeared to be similar in shape to the experimental EMG across conditions over the hop cycle, although the simulated muscle activations appeared to be time-delayed compared to the experimental EMG (figure 1). On average, similar *R^2^* values were found on a group- and individual-level (G - 0.41; I - 0.38) between experimental and simulated peak muscle activations (table 1; electronic supplementary material, S1). VL tended to have the highest *R^2^* values (G - 0.97; I - 0.87), followed by SOL (G - 0.60 ; I - 0.44).

**Table 1.** Group- and individual-level coefficients of determination (*R*^2^) between experimental and simulated energetics and muscle mechanics.

measures	*R*^2^	
	group	individual
peak SOL muscle activation	0.60	0.44
peak GL muscle activation	0.36	0.24
peak GM muscle activation	0.22	0.44
peak TA muscle activation	0.02	0.11
peak VL muscle activation	0.97	0.87
peak RF muscle activation	0.27	0.23
peak BF muscle activation	0.45	0.30
absolute SOL fascicle shortening	0.99	0.93
absolute GL fascicle shortening	0.94	0.77
absolute VL fascicle shortening	0.98	0.86
mean SOL fascicle shortening velocity	0.89	0.83
mean GL fascicle shortening velocity	0.72	0.63
mean VL fascicle shortening velocity	0.94	0.74
gross metabolic power	0.95	0.90

### 3.2. Muscle fascicle dynamics

**Figure 2.**
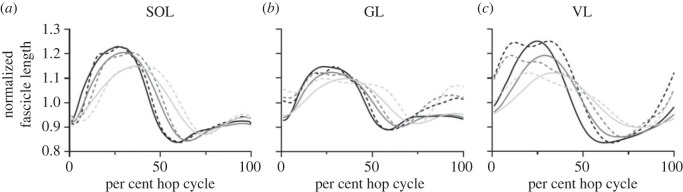
Time-series plots of the normalized experimental (dotted) and simulated (solid) fascicle lengths of three conditions (LH, MH, HH—from lightest to darkest) for SOL (*a*), GL (*b*), and VL (*c*). The data have been averaged across all participants for each condition. Experimental fascicle length is range normalized to the minimum and maximum value of the respective simulated fascicle length.

The simulated muscle fascicle dynamics appeared to be similar in shape and timing to the experimental data over the hop cycle (figure 2). On average, an *R*^2^ value of 0.97 was found for absolute fascicle shortening on a group-level, which weakened to 0.85 on an individual-level (table 1; electronic supplementary material, S2). Slightly weaker *R^2^* values were seen on average for mean fascicle shortening velocity on a group- and individual-level (G - 0.85; I - 0.73). SOL and VL had higher *R^2^* values than GL.

### 3.3. Metabolic power

**Figure 3.**
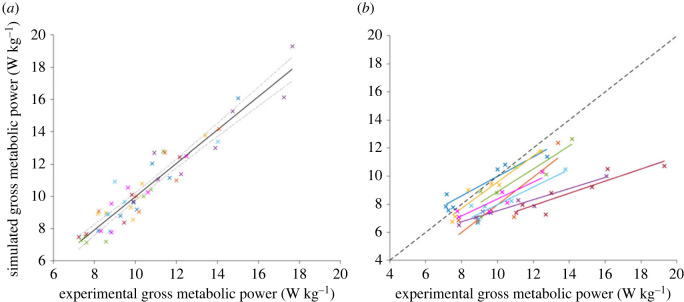
Depicting (*a*) linear model (including regression line (solid) and 95% confidence intervals (dotted)) from the individual-level analysis between experimental and simulated gross metabolic power (W kg^–1^), with different colours representing each individual participant's data; and (*b*) regression lines between each individual participant's original, non-fitted experimental and simulated gross metabolic power data.

Strong *R*^2^ values were found between experimental and simulated gross metabolic power on both a group- and individual-level (G - 0.95; I - 0.90) (table 1; figure 3*a*). There appeared to be some differences in intercept and slope for some participants when comparing the original, non-fitted experimental and simulated gross metabolic powers (figure 3*b*). Of the muscles that we tested, VL and SOL contributed the most to changes in simulated gross metabolic power between participants (18 ± 2% and 14 ± 1%, respectively). Equally, of the lower-limb muscle groups, knee extensors and ankle plantar flexors contributed the most to changes in simulated gross metabolic power between participants (30 ± 3% and 23 ± 2%, respectively).

## 4. Discussion

This study demonstrates the ability of computational musculoskeletal modelling to predict mechanical and energetic requirements across a range of movement conditions that are known to be mechanically and energetically disparate. Strong correlations were found between experimental and simulated peak muscle activations for the muscles that contributed substantially to the metabolic rate in each condition. Strong correlations were also found between experimental and simulated absolute fascicle shortening and mean fascicle shortening velocity for these same muscles. As such, strong correlations were found between experimental and simulated gross metabolic power. The correlations tended to be stronger on a group-level rather than individual-level. Therefore, while current modelling approaches seem adequate for distinguishing relative differences in gross metabolic power between hopping conditions, we identify several areas in which the accuracy of simulations could improve.

The correlations between simulated peak muscle activations and EMG signals were variable across muscles and seemingly depended on the variance measured across the conditions tested. Peak muscle activations showed the strongest correlations for VL and SOL. The correlations for other muscles tended to weaken across hop frequencies, rather than heights. This may be expected because force requirements change more drastically with changes in hop height. Moreover, compared to VL and SOL, the simulated activations of other muscles varied less between conditions. Thus, we feel that the inverse solutions from OpenSim Moco preferentially activated and handled uni-articular, extensor muscles best, compared to bi-articular and/or flexor muscles. The lower correlations may also be owing to the chosen objective function (i.e. minimizing the sum of square excitations) not being sufficient to capture subtle changes in control strategy.

One of the limitations that we had in comparing the experimental EMG to the simulated muscle excitations/activations was the time shifts that occurred because the activation dynamics in the model lacked an operable electromechanical delay term. Typically, an electromechanical delay in the range of 10–100 ms would be used [35–38]. Upon inspection of our data in real time, delays in this range would have accounted for the time shifts observed in figure 1. Notably, these time shifts happened to vary within and between muscles across the hopping conditions. While the results from the regression analyses performed in this particular study (table 1) are unlikely to have been affected by lack of an electromechanical delay, it is important that future models feature realistic excitation-activation dynamics. Despite the resultant time shifts, the simulated muscle activations appeared to do a good job of capturing the general shapes of the experimental EMG.

We observed good agreement between experimental and simulated fascicle dynamics. Moderate to strong *R^2^* values were found for absolute fascicle shortening and mean fascicle shortening velocity, and the experimental and simulated fascicle dynamics bared strong resemblances in both shape and timing. This gives us confidence that the muscle dynamics are sufficiently represented in the model. Since SOL and VL had far stronger correlations for peak muscle activations compared to GL, we were not surprised to find that these muscles also had stronger correlations for fascicle dynamics—activation dynamics and joint kinematics are used to model fascicle dynamics. Nevertheless, we find it interesting that these *R^2^* values remained moderate to strong despite levels of inaccuracy in the simulated muscle activations.

**Figure 4.**
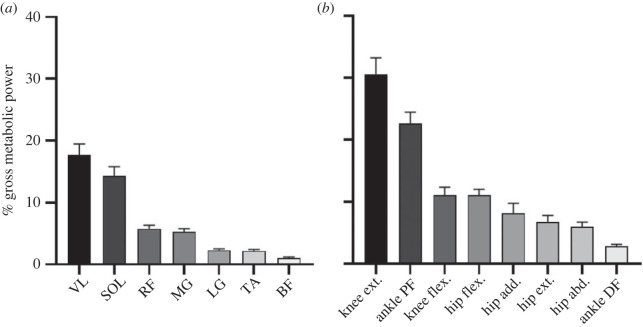
Mean ± s.d. contribution (%) of each muscle that we tested (*a*) and major lower-limb muscle groups (*b*) to changes in simulated gross metabolic power. The data have been averaged across all conditions for each participant.

Despite varying levels of correlation between experimental and simulated activation and fascicle dynamics, we found strong *R*^2^ values for gross metabolic power. These values, being 0.95 and 0.90 on a group- and individual-level, respectively, fall within the range of repeated measures coefficient (*R*_rm_) values found by Koelewijn *et al*. [16] across walking conditions (*R*_rm_ = 0.90–0.95). We presume that the strong correlations for gross metabolic power pertain to the fact that we found the highest *R*^2^ values in muscle mechanics for those muscles that contributed most to differences in simulated metabolic power between hopping conditions (i.e. SOL and VL) (figure 4). We are reluctant to comment on the absolute performance of the Umberger2010MuscleMetabolicsProbe given that the musculoskeletal model that we used was comprised lower-limb muscles only, and thereby ignored active metabolic costs owing to the trunk and arms. However, the differences that we found in intercept and slope between each participant's unadjusted experimental and simulated metabolic data indicate that simulations could not fully account for individual differences in basal metabolic rate and/or costs of performing mechanical work, respectively. Regardless, our results suggest that the approach that we took was sufficiently accurate for predicting increases and decreases in metabolic power across hopping conditions.

In general, we found that the *R*^2^ values between experimental and simulated data were considerably higher on a group- compared to individual-level. For example, across our subjects, *R*^2^ values for gross metabolic power ranged from 0.73 to 0.93, with a median of 0.81. Few model validation studies have presented individual-level insights, however Delabastita *et al*. [11], even after individualizing calf muscle-tendon parameters in their models, found that *R^2^* values for GM fascicle length changes in walking ranged from 0.26 to 0.84. Thus, although studies tend to report an improvement in the fit between experimental and simulated mechanics and energetics using subject-specific parameters [12,13,39], current modelling approaches may not be suitable for predicting muscle mechanics and energy expenditure for certain subjects/patients/athletes, which could be owing to factors such as training status, muscle fascicle lengths and volumes, tendon stiffnesses, or other anthropometrical differences (e.g. limb-mass proportions). Direct use of EMG signals in the tracking solutions (i.e. EMG-driven models) would probably have improved cost estimates associated with the activation of the muscles tested, however it is unclear whether such tuned models would still adequately represent the muscle dynamics or estimates of metabolic rate. Nevertheless, it is clear that the approach taken in this study, which only requires measures of kinematics and kinetics, performed adequately across the range of conditions tested.

We acknowledge that our results may have differed had we opted for a different muscle model or optimization software. The use of a Hill-type muscle model aided computational efficiency at the expense of capturing finer details of the morphology and interaction of muscle and tendon [40]. Moreover, we provide no assessment of the absolute performance of the modelling, which would have been limited given our use of a model of only lower extremity muscles. We further recognize that our results are contingent on the quality of our experimental data. Errors associated with our experimental measures are well-documented [1,23,27,41,42], and extreme care was taken when collecting and processing our data to follow directions of previous literature. However, if not a result of experimental error, we caution whether normalizing metabolic power exclusively to body mass contributed to differences in slope seen in figure 3*b*—our lightest participant (i.e. who weighed 46 kg (26 kg lighter than the sample mean)), had metabolic rates (shown in red) that diverged the most from the rest of the sample despite no apparent divergence in muscle mechanics. Lastly, it is worth noting that there is risk that we overestimated correlations since only seven conditions were fed into regression analyses for each participant.

To conclude, we used popular musculoskeletal modelling tools to predict mechanical and energetic demands across a range of human hopping conditions. In comparing the modelled outputs to our experimental data, we found strong correlations for peak activations in muscles contributing most to the metabolic rate (i.e. ankle and knee extensor muscles), strong correlations for fascicle shortening and shortening velocities in these same muscles, and strong correlations for gross metabolic power. The correlations for muscle activation and fascicle dynamics tended to weaken for flexor and biarticular muscles. Moreover, all correlations tended to be stronger on a group- than individual-level. Thus, current modelling approaches may be sufficient for predicting relative differences in metabolic power across movement conditions on a group-level, but caution is required for interpretation of simulation outputs for individuals. We also urge that appropriate validation be performed before running any analysis of simulated muscle mechanics, especially of muscle activations. To improve these predictions in relative and absolute terms, we encourage the community to use our dataset [43] and develop others like it to experiment with different musculoskeletal models, muscle models, metabolic cost models, optimal control policies, modelling tools and algorithms, data filtering etc., with personalized/subject-specific simulations being a focal goal.

## Data Availability

Jessup LN, Kelly LA, Cresswell AG, Lichtwark GA. 2023 MSK model validation dataset. *SimTK*. (doi:10.18735/DPCN-5P69) [43]. To access the dataset, go to *Downloads* > *Data Share* > *View*, click on the Study Title (*MSK model validation dataset*) and enter your SimTK login details. The data are provided in the electronic supplementary material [44].
